# Attributes influencing parental decision-making to receive the Tdap vaccine to reduce the risk of pertussis transmission to their newborn – outcome of a cross-sectional conjoint experiment in Spain and Italy

**DOI:** 10.1080/21645515.2019.1571890

**Published:** 2019-04-15

**Authors:** Edouard Ledent, Giovanni Gabutti, Esther W. de Bekker-Grob, Juan Luis Alcázar Zambrano, Magda Campins Martí, María Teresa Del Hierro Gurruchaga, María José Fernández Cruz, Giuseppe Ferrera, Francesca Fortunato, Pierfederico Torchio, Giorgio Zoppi, Christian Agboton, Walid Kandeil, Federico Marchetti

**Affiliations:** a Statistical Solutions & Innovations, GSK, Rixensart, Belgium; b Department of Medical Sciences, University of Ferrara, Ferrara, Italy; c Section Health Technology Assessment and Erasmus Choice Modelling Centre, Erasmus School of Health Policy & Management, Erasmus University Rotterdam, Rotterdam, The Netherlands; d Department of Obstetrics and Gynecology, Clínica Universitad de Navarra, Pamplona, Spain; e Servicio de Medicina Preventiva y Epidemiología, Hospital Universitario Vall d’Hebron, Barcelona, Spain; f Facultad de Medicina y Enfermería, Universidad del País Vasco, Leioa, Spain; g Hospital Universitario de Getafe, Getafe, Spain; h Centro de Salud Pintores, Parla, Spain; i Dipartimento Medico di Prevenzione-Servizio Epidemiologia, ASP Ragusa, Centro Servizi, Ragusa, Italy; j Department of Medical and Surgical Sciences, University of Foggia, Foggia, Italy; k Servizio Igiene e Sanità Pubblica, Cuneo, Italy; l Dipartimento di Prevenzione, Struttura Complessa Igiene e Sanità Pubblica, Chiavari, Regione Liguria, Italy; m Medical Affairs, GSK, Wavre, Belgium; n Global Medical Affairs, GSK, Wavre, Belgium; o Direzione Medica Vaccini, GSK, Verona, Italy

**Keywords:** Pertussis, vaccination, cocooning, Spain, Italy, adaptive choice-based conjoint questionnaire, adaptive discrete-choice experiment, preferences, Sawtooth software, survey

## Abstract

Pertussis vaccination of parents and household contacts (‘cocooning’) to protect newborn infants is an established strategy in many countries, although uptake may be low. Many aspects may influence such decision-making. We conducted a cross-sectional survey (NCT01890447) of households and other close contacts of newborns aged ≤6 months (or of expectant mothers in their last trimester) in Spain and Italy, using an adaptive discrete-choice experiment questionnaire. Aims were to assess the relative importance of attributes influencing vaccine adoption, and to estimate variation in vaccine adoption rates and the impact of cost on vaccination rates. Six hundred and fifteen participants (Spain, n = 313; Italy, n = 302) completed the survey. Of 144 available questionnaire scenarios, the most frequently selected (14% of respondents in both countries) were infant protection by household vaccination at vaccination center, recommendation by family physician and health authorities, with information available on leaflets and websites. The attribute with highest median relative importance was ‘reduction in source of infection’ in Spain (23.1%) and ‘vaccination location’ in Italy (18.8%). Differences between other attributes were low in both countries, with media attributes showing low importance. Over 80% of respondents indicated a definite or probable response to vaccine adoption (at no-cost) with estimated probability of adoption of 89–98%; applying vaccine costs (25€ per person) would reduce the probability of uptake by 7–20% in definite/probable respondents. Awareness of these determinants is helpful in informing Health Authorities and healthcare practitioners implementing a cocooning strategy for those populations where maternal immunization is not a preferred option.

## Introduction

Although effective vaccines against pertussis disease (caused by *Bordetella pertussis*) are available, with widespread implementation in childhood immunization programs, the incidence of this disease has increased in recent years.^1,2^ Epidemiological studies report a shift in the relative incidence from school-age children to adolescents and adults including older adults, in whom pertussis immunity (natural or vaccine-induced) has waned, and towards newborns and unvaccinated younger infants, in whom pertussis morbidity and mortality is greatest.^2-4^

Pertussis induces substantial direct medical costs among European countries.^5–7^ In Catalonia (Spain), total direct healthcare costs in children with pertussis aged 0–9 years in 2012–13 exceeded 700,000€^5^ while in Portugal, total costs for pertussis hospitalizations were higher than 2,500,000€ from 2000 to 2015.^6^

Maternal immunization is the most effective strategy to reduce the risk of pertussis in newborns.^2,8^ Nevertheless, alternative strategies are important when maternal immunization is declined. Protection of unvaccinated newborns via cocooning, where parents and other household contacts at risk of infection are vaccinated so forming a shield against newborn infection is an important strategy, especially in the absence of maternal immunization or as an additional complementary approach when disease prevalence is high.^8-11^

Cocooning has been recommended in the United States since 2006 while in Europe, cocooning has been recommended in a number of countries (e.g., France, Switzerland, and Germany) and may also be recommended following acute pertussis outbreaks (e.g., in Australia).^8,12-17^ However, implementation is highly variable and vaccine uptake by parents and other close contacts of newborns is often incomplete. For example, in France, although parental immunization (especially in mothers) has increased in recent years since the implementation of a national policy in 2004, a recent study reported that immunization of both parents was found in only 26% of families with infants aged <12 months.^13^ Data from Switzerland indicates that in the years immediately following implementation of cocooning in 2011, only 23% of mothers and 17% of fathers of newborns were vaccinated, and vaccination of all close-contacts was seen in only 7% of households with newborns.^15^

A range of determinants (or attributes) that influence vaccine uptake/acceptance by parents and household contacts have been reported, including risk of transmission to an infant, vaccine access and cost, healthcare professional (HCP) recommendations and disease awareness.^18-23^ Key determinants may vary in different countries and in different target groups (e.g., mother and partner), and understanding these aspects are important in developing and implementing a successful cocooning strategy.

In Spain, pertussis maternal immunization is well implemented in all regions (Catalonia was the first region to implement it in January 2014) with uptake around 80%^24^ while in Italy, maternal immunization is also recommended (and free of charge). In both countries, cocooning has been proposed as an adjunctive preventive strategy.^25-27^ To inform policy and determine which attributes of a pertussis cocooning vaccination strategy are important in parental decision making, we conducted a cross-sectional web-based survey in both countries, using an adaptive discrete-choice experiment (ADCE) questionnaire (also called an adaptive choice-based conjoint [ACBC] analysis) to measure preferences among households and other close contacts of newborns aged ≤6 months of age and among expectant mothers in their last trimester or their partners.

Discrete-choice experiments (DCE) are increasingly used to evaluate individuals’ vaccination preferences and underlying decision-making.^28-32^ While DCE allows participants to choose from a pre-defined set of attributes, an ADCE format allows more individualized choices, with successive choice scenarios adapted to previous questions in an interactive manner. In this ADCE approach, the respondent is first asked to identify their most ideal vaccination scenario, based upon key determinants that may influence choice (source of infection and likelihood of infection to the newborn, duration of protection, cost, vaccination location, recommendation by doctors or health authorities and supporting information and other opinions). For each respondent, the relative importance of each attribute is measured and ranked, and their personal preferences for specific attributes valued.

For the present study, the questionnaire was designed to minimize interaction between attributes i.e., to reduce the likelihood that preference for one attribute (or attribute level) affects the relative preference of another. This was achieved using the *Sawtooth Software* which uses a multinomial logit model and hierarchical Bayesian analysis when calculating part-worth utilities for each attribute and levels. Therefore, the survey questionnaire shows orthogonality between attributes (i.e., minimal correlation or interaction between attributes) and minimal overlap with level balance (i.e., each attribute appears only once in a choice set and attribute levels occur at an equal frequency within the questionnaire). In addition, the presence of any relevant co-linearity between model parameters was ruled-out following visual inspection of a scatter matrix presenting dependences between Monte-Carlo samplings of the joint posterior distribution of the parameters (Supplementary Figure 1).

**Figure 1.**
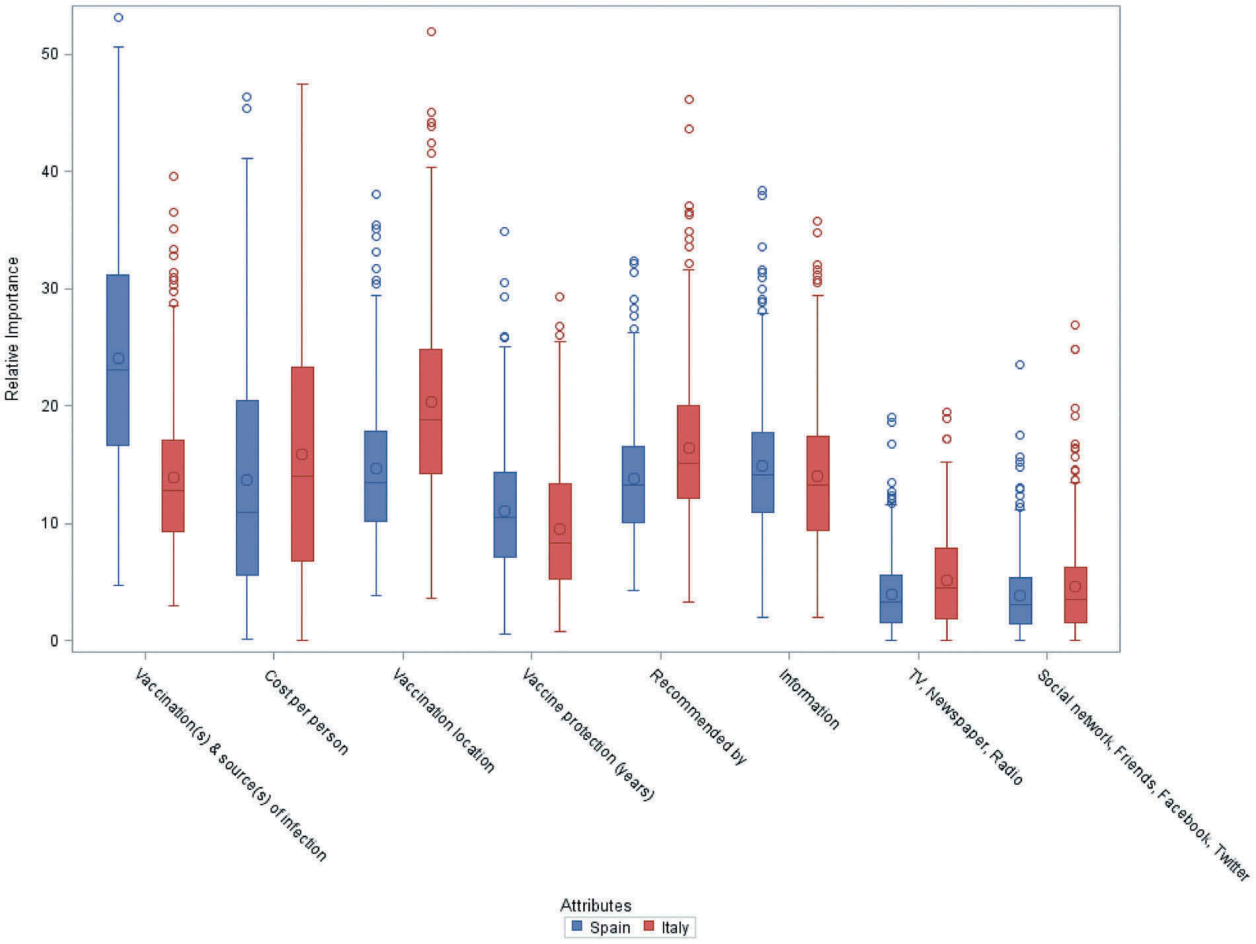
Relative importance of attributes. The distribution of the individual relative importance across subjects is presented for each attribute, separately for each country. The individual estimates of the relative importance are derived from the Bayesian estimations of the part-worth utilities using a hierarchical multinomial logit model. The limits of the boxes represent the 1st and 3rd quantiles and the bars in the middle represent the medians. The symbols within the boxes represent the means. The whiskers around the boxes extend up to 1.5 times the interquartile ranges. All extreme observations are shown using symbols beyond the whiskers.

The primary objective was to assess the relative importance of attributes that influence decision making in adopting pertussis vaccination by close contacts, with preferences evaluated within each country. Additional objectives included estimation of variations in pertussis vaccination adoption rates by contacts, the impact of vaccine costs on adoption rates, and to evaluate the role of demographic factors (e.g., gender, age) in choices and rates.

## Results

### Survey participants

Following a pilot questionnaire (scoped and developed based on focus group feedback), as described in the Methods section below, between January 2015 and February 2016 a total of 615 respondents completed the final questionnaire; 313 from Spain and 302 from Italy. Demographic characteristics of participants are shown in Table 1. Mean participant age (35 years) was similar in both countries and over 96% lived with their partner. Spanish participants were equally matched in terms of gender, while in Italy the majority of respondents (79%) were female. In Spain, 61.3% of respondents were expecting a child, while in Italy only 2.3% were expectant. Most participants already had children although the proportion was higher in Italy (89.4%) than in Spain (55.9%). While education levels were broadly comparable in either country, more Spanish respondents had a University degree than Italian respondents (Table 1).

**Table 1.** Characteristics of participants who completed the final study questionnaire.

Parameter	Spain (N = 313)	Italy (N = 302)
**Personal data**		
Age in years, mean (range)	34.9 (20.0–67.0)	35.9 (19.0–62.0)
Gender, n (%)		
Female	157 (50.2)	237 (78.5)
Male	156 (49.8)	65 (21.5)
Ethnicity, n (%)		
Caucasian	300 (95.8)	289 (95.7)
Arabic/North African	8 (2.6)	3 (1.0)
African/African-American	1 (0.3)	2 (0.7)
Other	4 (1.3)	8 (2.6)
Education level, n (%)		
Primary school	19 (6.1)	4 (1.3)
High school	81 (25.9)	153 (50.7)
Bachelor’s degree	59 (18.8)	48 (15.9)
University degree	96 (30.7)	65 (21.5)
Other	58 (18.5)	32 (10.6)
Living with a partner, n (%)	303 (96.8)	291 (96.4)
Already has children, n (%)		
1	114 (36.4)	135 (44.7)
2 or more	61 (19.5)	135 (44.7)
Expecting child, n (%)	192 (61.3)	7 (2.3)
**Pertussis experience**		
Personal or family member or social contacts with prior infection, n (%)	27 (8.6)	97 (32.1)
Previous pertussis vaccination, n (%)		
Yes	61 (19.5)	59 (19.5)
Yes, and within previous 10 years	3 (1.0)	14 (4.6)
Yes, but cannot recall when	28 (8.9)	30 (9.9)
No	102 (32.6)	135 (44.7)
Uncertain	150 (47.9)	108 (35.8)

Most participants had no prior direct experience of pertussis (either personal or within their family/social contacts), although perception of pertussis was more frequent in Italy than in Spain (32.1% and 8.6% respectively). In either country, less than 20% of patients could recall any prior pertussis vaccination and most that did so could not clearly recall when this was; the number reporting vaccination anytime within the previous 10 years was low; Spain (1.0%) and Italy (4.6%) (Table 1).

### Questionnaire experience

The questionnaire was well understood by most respondents, with 94.9% of Spanish respondents and 97.4% of Italian respondents understanding all of the survey questions; Spanish respondents took slightly longer in completion time than in Italy (21.1 and 15.2 minutes respectively). An example of a questionnaire choice is shown in Supplementary Figure 2.

**Figure 2.**
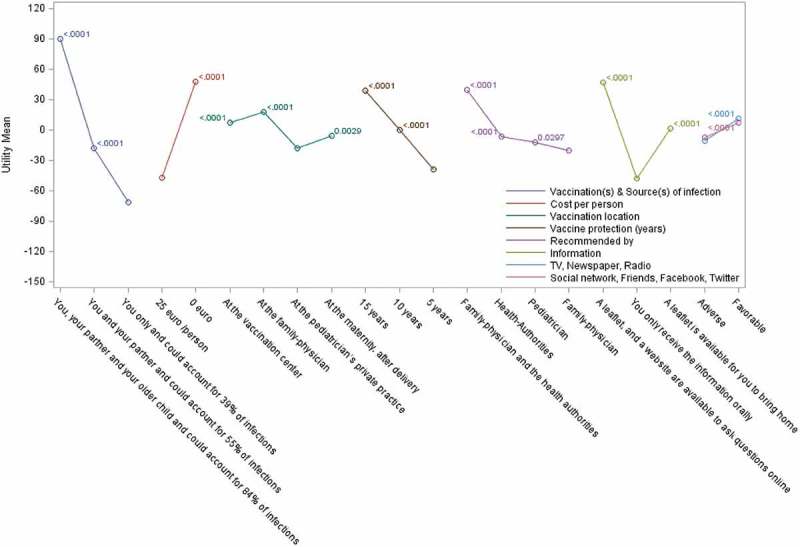
Relative utilities and utility differences in Spain. Mean part-worth utilities are presented using a colored line connecting all levels for the same attribute. Bonferroni-adjusted p-values for the difference compared to the utility of the lowest level are presented close to each point.

### Preferred scenarios for vaccine decision making

Participants built their preferred vaccination scenarios based upon key attributes included in the questionnaire (Table 2). Among the 144 scenarios fully available to the respondent, infant protection by household vaccination at a vaccination center, recommended by a family physician and health authorities, with information available on leaflets and websites, was the scenario most frequently selected (by 14% of the survey sample in both countries) (Table 3). In both countries, vaccine recommendation by both family physician and health authorities was important, along with supportive information provided both by leaflets and websites. The proportion choosing their partner and other children to be vaccinated in preferred scenarios was lower in Italy than in Spain. Italian participants favored immunization at vaccination centers, while many Spanish respondents opted for vaccine administration by their family physician or maternity unit.

**Table 2.** Attributes and level of attributes used for the formal study questionnaire.

Attribute	Levels
Vaccination(s) & source(s) of infection	You only and could account for 39% of infections
	You and your partner and could account for 55% of infections
	You, your partner and your older child and could account for 84% of infections
Cost per person	25 euro
	0 euro
Vaccination location	At the pediatrician’s private practice
	At the maternity unit, after delivery
	At the vaccination center
	At the family physician
Vaccine protection (years)	5
	10
	15
Recommended by	Family physician
	Pediatrician
	Health Authorities
	Family physician and the health authorities
Information	You only receive the information orally
	A printed leaflet is available for you to bring home
	A printed leaflet and a website are available to ask questions online
TV, Newspaper, Radio	Adverse
	Favorable
Social network, Friends, Facebook, Twitter	Adverse
	Favorable

**Table 3.** Most ideal vaccination scenario selected by at least 3% of the Spanish and Italian participants.

Vaccination(s) & source(s) of infection	Cost	Location	Duration of protection (years)	Recommended by	Information provided by	Media	Social network	n	%
**Spain (N = 313)**									
You, your partner and your older child and could account for 84% of infections	0€	Vaccination center	15	Family physician and health authorities	Printed leaflet and a website to ask questions online	Favorable	Favorable	46	14.7%
You, your partner and your older child and could account for 84% of infections	0€	Family physician	15	Family physician and health authorities	Printed leaflet and a website to ask questions online	Favorable	Favorable	34	10.9%
You, your partner and your older child and could account for 84% of infections	0€	Maternity unit, post-delivery	15	Family physician and health authorities	Printed leaflet and a website to ask questions online	Favorable	Favorable	23	7.3%
You, your partner and your older child and could account for 84% of infections	0€	Maternity unit, post-delivery	15	Pediatrician	Printed leaflet	Favorable	Favorable	13	4.2%
You and your partner and could account for 55% of infections	0€	Family physician	15	Family physician and health authorities	Printed leaflet and a website to ask questions online	Favorable	Favorable	12	3.8%
You, your partner and your older child and could account for 84% of infections	0€	Family physician	15	Family physician	Printed leaflet and a website to ask questions online	Favorable	Favorable	12	3.8%
You, your partner and your older child and could account for 84% of infections	0€	Maternity unit, post-delivery	15	Pediatrician	Printed leaflet and a website to ask questions online	Favorable	Favorable	11	3.5%
You, your partner and your older child and could account for 84% of infections	0€	Family physician	15	Family physician and health authorities	Printed leaflet	Favorable	Favorable	10	3.2%
**Italy (N = 302)**									
You, your partner and your older child and could account for 84% of infections	0€	Vaccination center	15	Family physician and health authorities	Printed leaflet, and a website to ask questions online	Favorable	Favorable	42	13.9%
You and your partner and could account for 55% of infections	0€	Vaccination center	15	Family physician and health authorities	Printed leaflet and a website to ask questions online	Favorable	Favorable	12	4.0%
You only and could account for 39% of infections	0€	Vaccination center	15	Family physician and health authorities	Printed leaflet and a website to ask questions online	Favorable	Favorable	12	4.0%
You, your partner and your older child and could account for 84% of infections	0€	Vaccination center	15	Health authorities	Printed leaflet and a website to ask questions online	Favorable	Favorable	11	3.6%
You and your partner and could account for 55% of infections	0€	Vaccination center	15	Pediatrician	Printed leaflet and a website to ask questions online	Favorable	Favorable	11	3.6%
You and your partner and could account for 55% of infections	0€	Vaccination center	15	Health authorities	Printed leaflet	Favorable	Favorable	10	3.3%

Respondents were asked to select their preferred option for each of the 4 attributes for which options were not offered logically (‘Vaccination and source(s) of infection’; ‘Location’; ‘Recommended by’; and ‘Information provided by’. For other attributes, respondent choice was imputed based upon the best logical answer (Cost, €0; Duration, 15 years; Media, favorable; Social network, favorable).

### Relative importance of attributes that influence vaccine decision making

The relative importance of individual attributes varied between countries. The attribute showing the highest relative importance for Spanish respondents was the reduction in source of infection (median, 23.1%; mean, 24.0%) with other criteria of less importance (Figure 1 and Supplementary Table 1). In Italy, the differences in relative importance between attributes were low, with median values mostly in the range of 8.5–15%, with the highest importance assigned to vaccination location (median, 18.8%; mean 20.4%). In both countries, the two attributes reflecting perception of media and social contacts scored very low in relative importance (median values, Spain, 3.1% and Italy, 3.5%).

When ranking the individual relative importance (calculated for each participant), a slight majority of Spanish respondents (50.2%) selected ‘vaccination(s) & source(s) of infection’ as the most important attribute, while only 9.9% of Italians did so (Supplementary Table 2). In contrast, vaccine administration location (28.8%) and vaccine cost (26.8%) were selected as having highest individual relative importance by Italians. Overall, based on respondent’s individual relative importance estimates, the number of instances each attribute was selected as the most important differed significantly between countries (Chi-squared test, P < 0.01).

The number of instances each attribute was selected as one of the three most important attributes, based on each respondent’s relative importance, is shown in Supplementary Table 3. Comparisons between specific respondent subgroups did not show any significant association with participant gender (with p-values of 0.08 and 0.21 for Spain and Italy respectively). Similarly, no apparent associations between participants’ level of education or participant age (i.e., between those <35 years and ≥35 years old) and attribute ranking were observed (data not shown). The study, however, was not powered to demonstrate specific differences in subgroups between or within countries.

### Preferences among each attribute

**Figure 3.**
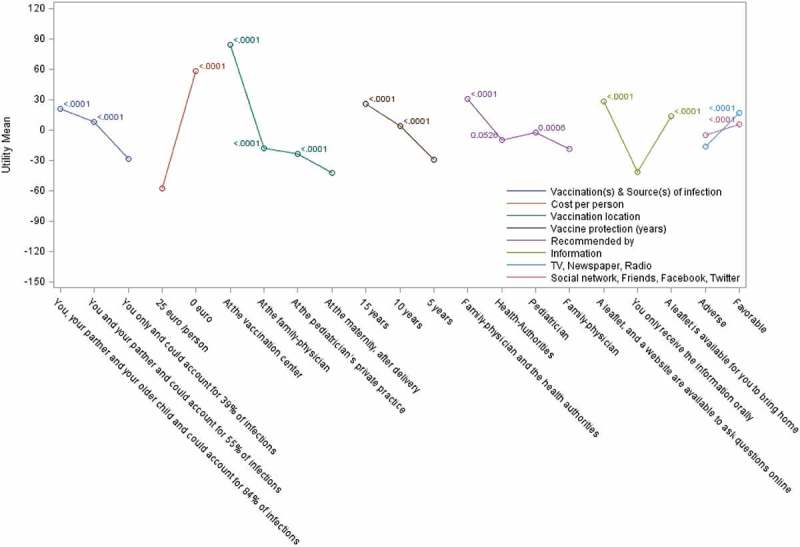
Relative utilities and utility differences in Italy. Mean part-worth utilities are presented using a colored line connecting all levels for the same attribute. Bonferroni-adjusted p-values for the difference compared to the utility of the lowest level are presented close to each point.

For each attribute, we analyzed the perceived value of the various options proposed (with adjustment for multiple-comparisons using the Bonferroni method). For most attributes, the choice of lowest attribute preference and of attribute preference order was identical in both countries (Figures 2 and 3 and Supplementary Table 4). Exceptions were for ‘vaccination location’, where ‘pediatrician’s private practice’ was least preferred in Spain and vaccination at the ‘maternity unit post-delivery’ was least preferred in Italy, with minor changes to the ranking of recommendation source. Ranking of ‘vaccination(s) & source(s) of infection’ options showed a consistent preference for higher level of reduction in source of infection, together with a higher number of contacts being vaccinated. The availability of leaflets and websites as sources of information was preferred in both countries over traditional oral communication (which was least preferred). Having both family physicians and health authority entities recommending vaccination consistently favored vaccine adoption. Among each attribute, the various options chosen were statistically superior to the least preferred option used as the reference, with the only exception being the ‘Health authorities’ recommendation source in Italy (P = 0.05) with ‘Family physician’ attribute as reference.

**Table 4.** Participant’s subjective opinions on vaccine adoption under their most preferred scenario.

Country^a^	Definitely will not buy		Probably will not buy		Might or might not buy		Probably will buy		Definitely will buy	
	n	%	n	%	n	%	n	%	n	%
Spain (N = 311)	5	1.6%	4	1.3%	21	6.8%	83	26.7%	198	63.7%
Italy (N = 297)	3	1.0%	10	3.4%	44	14.8%	124	41.8%	116	39.1%

Respondents were presented scenarios based upon their previous survey responses, as included in their preferred scenarios. Data presented corresponds to the likelihood of vaccination adoption for that scenario. A Likert-scale was used to capture respondents’ readiness to ‘buy’ (i.e., adopt) the proposed vaccination option.

^a^Some subjects did not complete this section of the survey (Spain, n = 2; Italy, n = 5).

### Vaccine adoption

**Figure 4.**
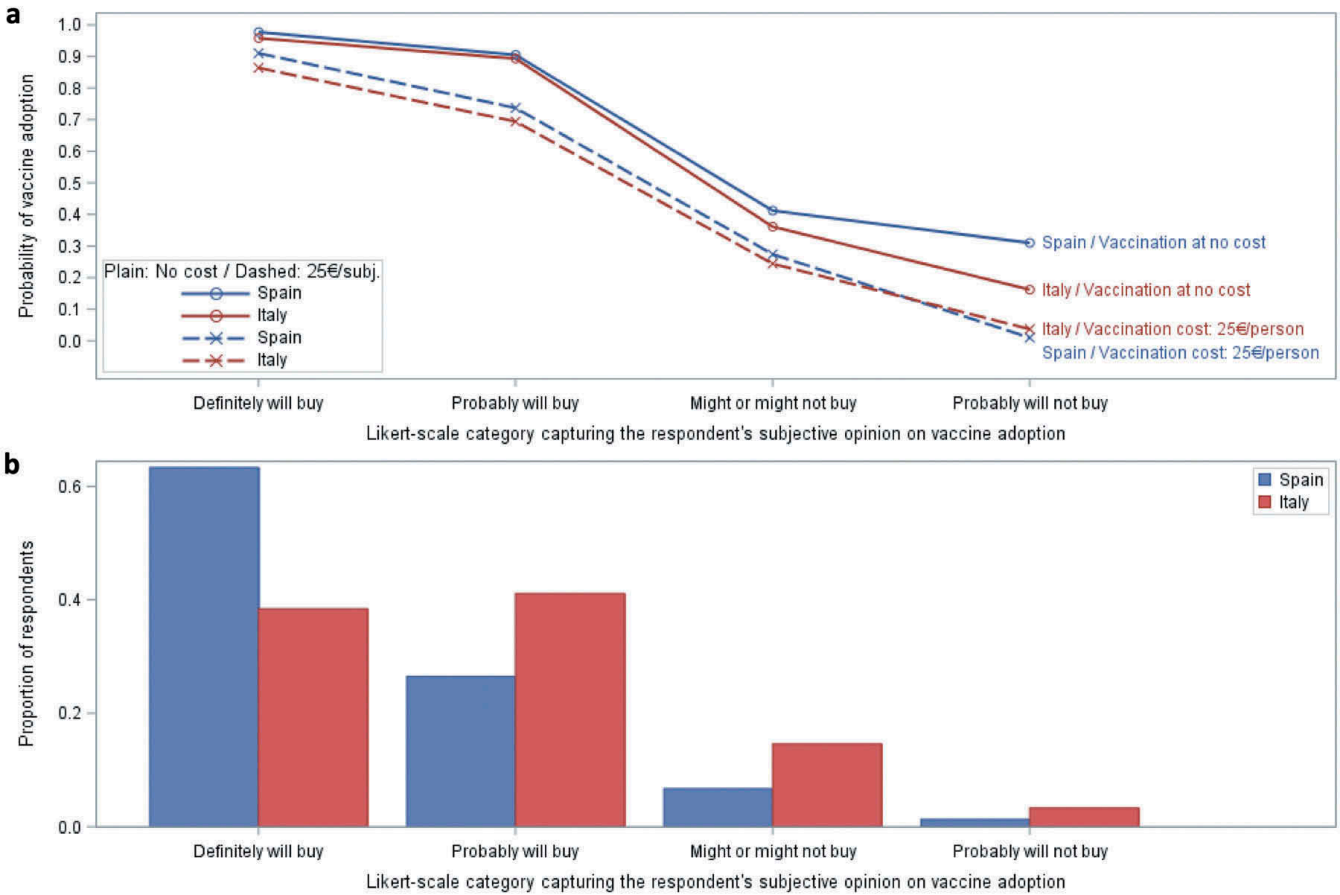
Probability of vaccine adoption. Figure 4(a) represents the respondent probability of vaccine adoption using logistic regression calibrated through a series of six vaccination scenarios. Figure 4(b) represents the proportion of respondents choosing possible likelihood of adoption level preferences. Probability of adoption is calculated considering the most ideal vaccination scenario for each subject assuming vaccination at zero cost (solid curve) and at a cost of 25€ per person (dashed curve) presented for both Spain and Italy. Probabilities are based upon the subjective opinions of the respondents on their likelihood of adoption under their ideal vaccination scenario. The ‘Definitely will not buy’ category included in Supplementary Table 5 is not presented graphically as the number of subjects contributing to that category was not sufficient to provide reliable estimates.

The proportion of participants choosing probable or definite vaccine adoption was high (Table 4 and Figure 4). When asked about their subjective opinion on adoption under their preferred (zero cost) vaccination scenario (as rated on a 5-point Likert-scale), a majority of respondents in both Spain (90.4%) and Italy (80.8%) indicated a definite or probable adoption response, although a larger proportion of Spanish participants definitely would adopt vaccination (63.7%) than in Italy (39.1%).

The estimated probability of vaccination adoption under their preferred (zero cost) vaccination scenario was 97.7% for Spanish respondents and 95.8% for Italian respondents (Supplementary Table 5). Vaccine costs had an impact on adoption. When vaccine costs (25€ per person) were applied, the probability of uptake fell by 7–10% in those participants initially choosing to definitely adopt; from 97.7% to 91.0% in Spain and from 95.8% to 86.4% in Italy. In those choosing to probably adopt, the probability of uptake also fell (from 90.5% to 73.7% in Spain and from 89.3% to 69.4% in Italy). For those participants who were less certain, a 25€ vaccine cost lowered adoption rates by 11–30% (Supplementary Table 5 and Supplementary Figure 3). These reductions in the probability of uptake in the various Likert-scale categories are the consequence of the preferences for zero cost vaccination (Figure 4).

## Discussion

Understanding key determinants of decision-making is an important aspect of vaccine policy and implementation to counteract vaccine hesitancy. While previous cross-sectional questionnaire studies have evaluated parental personal and psychosocial determinants of cocooning adoption,^20,21^ to our knowledge this is the first study formally evaluating broader aspects of pertussis cocooning using a conjoint approach.

An adaptive approach to DCE is an established method in evaluating healthcare choices,^33-36^ and comparisons between adaptive and more standard DCE approaches show comparable results,^37-39^ with the adaptive approach allowing screening of a larger number of attributes and levels with a lower number of subjects. ADCE also limits some bias.^40^ An adaptive implementation (i.e., ADCE) was considered to provide a better fit to the aims of our surveys than a DCE approach; mainly due to the greater amount of individualized data collected and the lower variance offered by the ADCE approach compared with that obtained from a static DCE approach. Attribute levels were selected based upon vaccination and implementation policy, with data for the relative proportion of infant infection due to specific types of household contacts based on that reported by Wiley et al.^41^

Our survey indicates that the uptake of pertussis vaccination to provide indirect protection to their newborn infant in a cocooning strategy was positively received by mothers and fathers in both Spain and Italy, with 89–98% indicating a definite or probable adoption response. This is consistent with previous studies exploring attitudes to cocooning, with surveys conducted in Dutch and Australian populations reporting positive intention rates of 78% and 96% respectively.^20,21^ The respondents’ most frequent preferred scenario was one that offered the greatest protection; vaccination of all household contacts (i.e., themselves, their partner and older children), an aspect also supported by Dutch and Australian studies, where risk perception (of pertussis disease and transmission to infant) and expected efficacy of the pertussis vaccination were key themes in forming a positive opinion to cocooning, with such perceived vaccine benefits being an independent predictor of vaccine uptake.^19,21,22^

Vaccination habit seems to have an important influence on respondent preferences. In Italy, greater preferences seem to be given to vaccination centers, which are well established in delivering disease-prevention measures for all ages. However, in Spain, vaccination settings are different: children receive vaccinations in primary care centers by a pediatric nurse assigned to the child, whereas adults are administered vaccinations by a public health nurse assigned to the adult. Due to these organizational differences between Spain and Italy, the importance of preference location and specific location of delivery was more variable between the two countries.

The majority of our survey respondents in both countries had limited perception or experience of pertussis and vaccine recommendations by both their family physician and the health authorities were important factors in their decision-making, along with information about benefits (both printed information and that available on a website). In comparison, the value of other media information or that from social contacts were considered less important. The value and source of professional recommendations in influencing adoption of vaccination in general and of pertussis cocooning in particular has been reported in a number of studies,^14,18-20,26,42-45^ In China, vaccination promotions have been shown as influencing parent attitudes toward children vaccination^44^ and, in sub-Saharan Africa, access to information has been highlighted as being a key determinant in reducing the rates of missed opportunities for children immunization.^45^ In Australia, one study found that willingness to receive pertussis vaccination post-partum was 7-fold greater in women who had received a recommendation compared to those without any such information,^43^ and a second Australian study found that acceptance was 1.7 times more likely following a recommendation.^19^ Studies performed elsewhere e.g., in the United States, report even greater influences of professional recommendations on adoption of pertussis cocooning.^46^

We found some differences in the relative importance of attributes in either country (e.g., reduction in infection risk was more important to Spanish respondents, whereas location of vaccine delivery was more important to Italian respondents). Notably, gender did not appear to influence the relative importance of these determinants on vaccination choices.

Cocooning prevention is less cost effective than maternal immunization, as a high reduction in the numbers of sources of infection requires vaccinating all household contacts and potentially other newborn-contacts.^47^ As such, cocooning should be considered when maternal ante-partum vaccination should be avoided or when it is not the mother’s personal preference.

Data on the impact of vaccine cost on the adoption of a cocooning strategy by close contacts is limited. In our survey, we found that including a vaccine cost (of 25€ per person) had a relatively minor effect on the probability of vaccine adoption in the most convinced ‘Definitely will buy’ respondents (63.7% and 39.1% of Spanish and Italian respondents respectively). In those subjects that answered ‘Probably will buy’ a reduction in the mean probability of 16–20% was observed in that group. Vaccination cost is therefore an important factor in vaccine adoption, especially in those that have some hesitations or doubts; 26.7% and 41.8% of all respondents in Spain and Italy respectively.

In Italy, the tetanus-diphtheria-acellular pertussis decennial booster vaccine (Tdap) for adults and elderly is now included free of charge as part of the National Immunization Program. In view of our survey results, an increase of Tdap acceptance by a broader group of eligible adults could be realized in Italy, provided that other important determinants (i.e., place of vaccination, recommendation by physicians and authorities, etc.) are fulfilled. In contrast, in Spain, Tdap is provided at no cost only to pregnant women and healthcare workers, and so uptake by other adult close contacts may be influenced by cost.

Our study has some limitations. Participant recruitment makes the survey results vulnerable to selection bias. Our survey was not designed to be representative of the Spanish or Italian populations and reflects opinions of a random sample of subjects visiting the investigating centers (and subjects who were not *a-priori* against vaccination as a prevention measure). Our principal aim was to evaluate vaccine preferences qualitatively within each country, and pooled across both countries if no substantial differences between Spain and Italy were found. The statistically significant differences we found between countries did not allow us to do so. While we have reported some of these differences in preferences, these should be considered in the context of a relatively small sample size in a non-randomized cohort.

Some demographic differences between Spain and Italy may have influenced the differences we observed in attribute ranking (and indeed differences between centers within each country). For example, the majority of Italian participants already had newborn contact and so we were unable to evaluate the choices of expectant newborn contacts in Italy, and indeed some attribute levels (e.g., vaccination at the maternity unit) were therefore irrelevant and hence given a lower preference score. As described above, differences in vaccine services and delivery locations may also influence differences between countries. In addition, we did not formally evaluate differences based on the relationship of the respondent (i.e., mother, father, or another adult household contact) to the newborn or expected newborn. Other demographic variations (including respondent education level, income and nature of recruitment center) could also have influenced our results, as our study design and sample size did not allow stratification of results for specific analysis of these aspects.

Finally, although a high level of internal consistency was seen during scoping and survey development, the questionnaire was not formally externally validated according to a formal vaccine buying and administration process.

Nevertheless, our survey identifies key aspects that influence cocooning acceptance by parents and close contacts of Spanish and Italian newborns, and demonstrates a high rate of positive intention of pertussis vaccine uptake.

**Figure 5.**
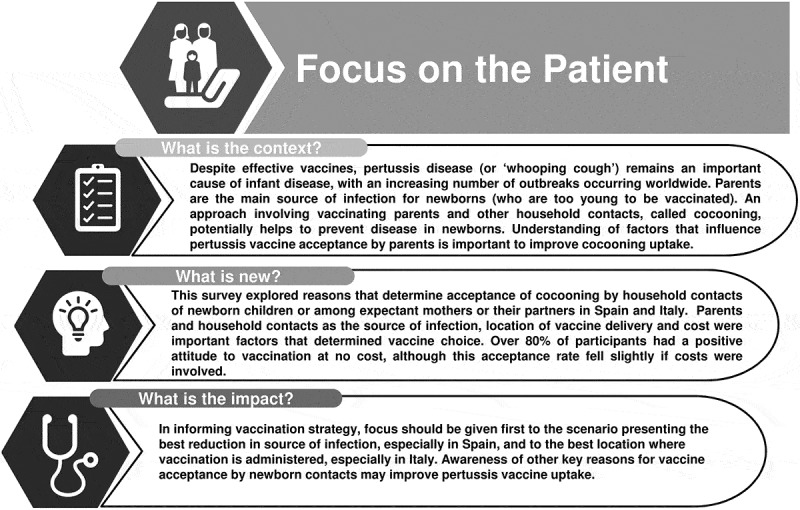
Focus on the patient section.

Figure 5 summarizes the context, outcomes, and impact of this survey for HCPs. Our survey was intended to explore factors influencing vaccine uptake and vaccine hesitancy to better understand how to improve patient communication. Our survey suggests that vaccine adoption would be welcomed by those Italian and Spanish parents who do not harbor inherent objections to vaccination. Appreciating the attributes described in this survey that influence vaccine uptake may assist communicating future pertussis vaccination strategies as necessary. This information may be helpful in assisting HCPs in Spain and Italy to engage with their appropriate vaccine target populations.

## Methods

### Study design and study population

This was a cross-sectional questionnaire study (NCT01890447) conducted between January 2015 and February 2016 in 313 adults from five centers in Spain (Barcelona, Getafe/Madrid, Leioa, Pamplona, and Parla) and in 302 adults from four centers in Italy (San Severo, Chiavari, Ragusa, and Cuneo). Participants were households or other close contacts of newborns aged ≤6 months of age (or expectant mothers in their last trimester or their partners), recruited from those receiving routine pre- or post-natal obstetric care. All participants had to be ≥18 years of age, capable of understanding the questionnaire, and had to be eligible to receive pertussis booster vaccination (with no history of pertussis vaccination in the previous 2 years). Individuals not fulfilling these criteria or those with self-declared objections to vaccination, those with a history of pertussis in the last 5 years before the start of the study, and those with unstable chronic health condition(s) were ineligible. No financial incentives were offered.

### Study questionnaire development

The study was performed in two stages; an initial pilot questionnaire design phase followed by a formal enrolment study phase. Before the pilot phase, draft questionnaire development was facilitated by a focus group approach.^48^ Three investigator-led focus groups (drawn from HCPs and parent representatives) were formed in each country (between September-December 2013) and a range of potential attributes to be considered for inclusion in the questionnaire were discussed, along with how to present attribute choices. All feedback was collated and centralized. The key attributes were identified and their level of importance formed the basis of a draft questionnaire, which included 1) an explanation of pertussis infection and the rationale for cocooning; 2) study objectives; and 3) a sequence of questions regarding reasons/attributes that influence vaccination choice and levels of importance. In general, the results from Spanish and Italian focus groups were consistent.

Both the pilot draft questionnaire and the final formal survey questionnaire were designed and implemented using the *Sawtooth Software* SSI-Web version 8.4.8 (Sawtooth Software; SSI Web, Orem, Utah, United States). Identical questionnaires were administered in both countries; questionnaires were initially developed in English and then translated into Spanish and Italian language versions.

In the pilot phase, approximately 50 participants from each country were randomized (computer generated 1:1 ratio) to complete either the DCE or ADCE questionnaire (conducted between September 2014 and January 2015). Based on the pilot phase, an ADCE format was considered more suitable for the formal survey; in particular, as some subjects considered some attributes as unacceptable (suggesting non-compensatory behavior) we preferred to use a methodology that accounted specifically for such behavior. In addition, these attribute levels were further defined and updated with the final questionnaire showing near-complete orthogonality (i.e., minimal correlation between attributes).

### Final questionnaire

Demographic data (age, gender, education status, number of children, contacts at home, and pertussis vaccination history) were collected from each respondent completing the final questionnaire (between January 2015 and February 2016). The definitive questionnaire comprised questions on eight attributes; source of infection and likelihood of infection to the newborn; duration of vaccine protection; vaccine cost; vaccination location (primary care physician’s office, pediatrician’s office, vaccination center/clinic, in maternity unit following delivery); source of vaccination recommendation (i.e., pediatrician, primary care physician and/or health authority); source of supporting information (oral, printed leaflet and/or internet-based); and opinions expressed in traditional (newspapers, television/radio) and social media or by acquaintances. Each attribute was described in terms of distinct scenarios so generating a number of choice sets with two or more hypothetical options.

### Sample size and data analysis

Determining sample size in conjoint analyses is challenging,^49^ as this depends on the proportion of respondents choosing each attribute scenario. As this was unknown to us prior to enrolment, we adopted an incidence precision approach^50^ from which we anticipated that, for a minimum number of 8 choice sets per participant, a minimum of 200 individuals from each country was required to achieve 10% precision at a 95% confidence level. The sample size was increased to 250 to account for any non-minimal-variance in questionnaire design. In actuality, over 300 participants from each country were recruited.

All data was anonymized and the statistical analyzes were performed using the *Sawtooth Software* SSI-Web version 8.4.8 and *SAS* (version 9.4M2 on Windows). Data on participant demographics and attribute relative importance are presented in descriptive terms. The relative importance of attributes indicates the impact of that attribute in participant decision-making in choosing a vaccination scenario, relative to all other attributes. Utility values (‘part-worth’ values) for each attribute were calculated using the part-worth utilities of the multinomial logit model and hierarchical Bayesian analysis implemented by the *Sawtooth Software*. Utility differences were calculated between the most and the least preferred options for each attribute, with the relative importance based on the extent of these utility differences. The relative importance for each attribute was calculated for each participant, leveraging hierarchical Bayesian analysis. Ranking of importance across attributes was based on the average relative importance values across the whole respondent population and the proportion of participants rating that attribute as having the highest relative importance. For preferences among specific attributes, the differences between the mean (‘part-worth’) utilities were calculated, using the lowest utility as a reference, with approximate p-values calculated after adjustment for multiple-comparisons by the Bonferroni method.

The impact of vaccine costs on the probability of vaccine adoption was calculated using relative utilities; the utility threshold above which the respondent would adopt vaccination was calibrated through a series of six vaccination scenarios and the stated (subjective) likelihood of adoption for each scenario. The utility threshold above which an adoption would occur was based on a regression approach. Likelihood of adoption was elicited using categories of subjective participant opinions; definitely/probably will not buy, might or might not buy, probably/definitely will buy, and utilities for which the subjective opinion was superior to ‘might or might not buy’ were considered to lead to adoption. A logistic regression curve was calibrated on these data, using a 50% probability of vaccination adoption at the approximate mid-point between ‘Might or might not buy’ and ‘Probably will buy’ levels of the Likert-scale used to capture each respondent’s answer. Probabilities of vaccine adoption were calculated for each participant’s most preferred scenario (zero cost) and compared to probabilities when a 25€ per person cost was applied.
