# Supplementary material for: Attributes influencing parental decision-making to receive the Tdap vaccine to reduce the risk of pertussis transmission to their newborn – outcome of a cross-sectional conjoint experiment in Spain and Italy
Source: Hum Vaccin Immunother. 2019 Apr 15;15(5):1080–91. doi: 10.1080/21645515.2019.1571890 (PMC6605846; doi:10.1080/21645515.2019.1571890)
Supplement: Supplemental Material [file khvi-15-05-1571890-s001.zip › Supplementary Figure 1_Assessment.docx]

# **Supplementary Figure 1. Assessment of co-linearity**

#


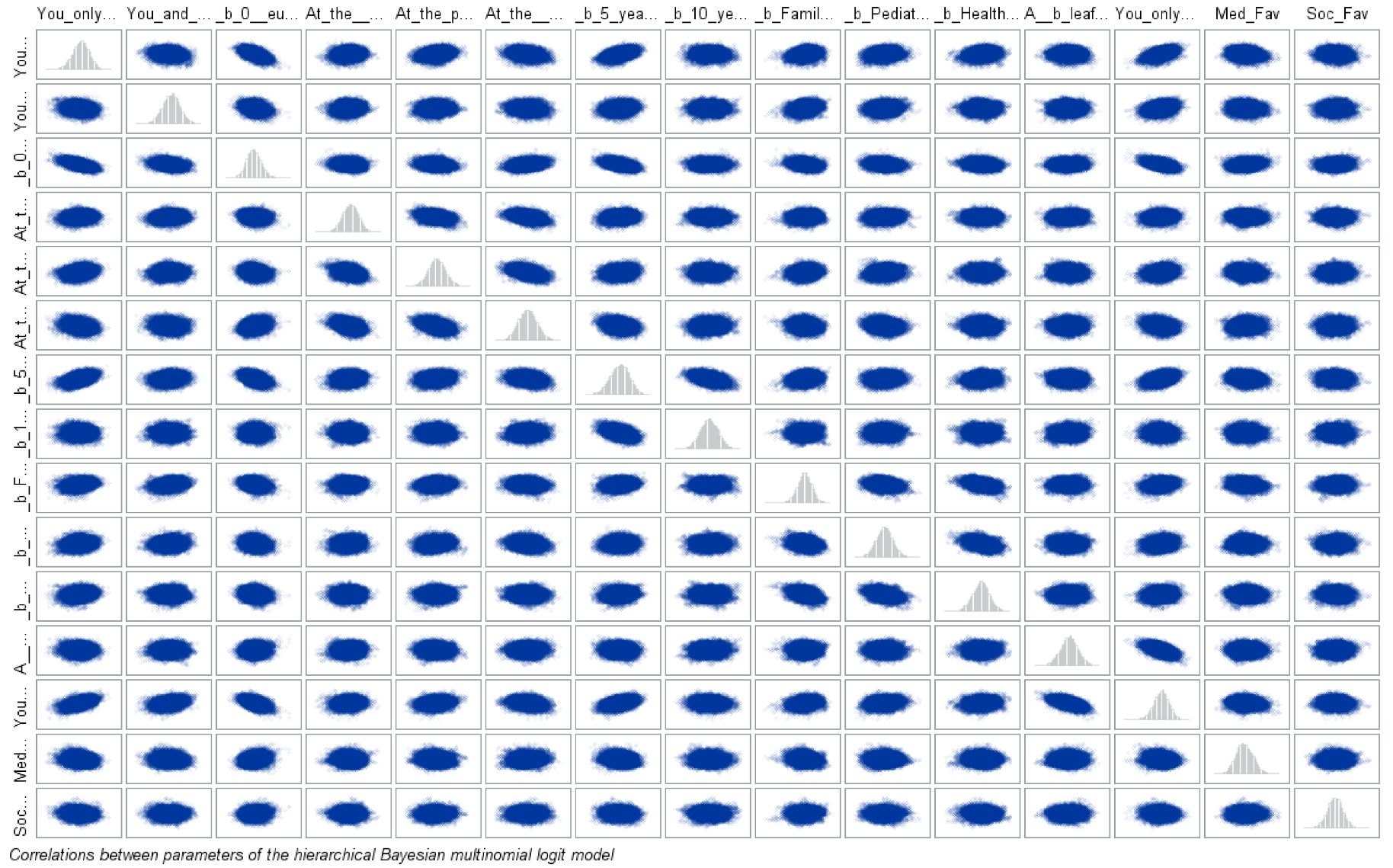


Diagnostic plot illustrating the absence of any relevant dependencies or co-linearity between part-worth utilities. The off-diagonal cells present the bivariate distribution of the x-axis and y-axis parameters included in the model. The diagonal histograms provide insights on the marginal distribution of each parameter. None of those bivariate distributions show extreme dependence as a consequence of co-linearity. Some bivariate plots present some level of correlations between the 2 parameters but exclude any co-linearity.
