# Supplementary material for: Attributes influencing parental decision-making to receive the Tdap vaccine to reduce the risk of pertussis transmission to their newborn – outcome of a cross-sectional conjoint experiment in Spain and Italy
Source: Hum Vaccin Immunother. 2019 Apr 15;15(5):1080–91. doi: 10.1080/21645515.2019.1571890 (PMC6605846; doi:10.1080/21645515.2019.1571890)
Supplement: Supplemental Material [file khvi-15-05-1571890-s001.zip › Supplementary Figure 2_ADCE.docx]

# **Supplementary Figure 2. Examples of a survey using the adaptive discrete choice experiment (ADCE) method**

1. **Identify the local maximum utility scenario**


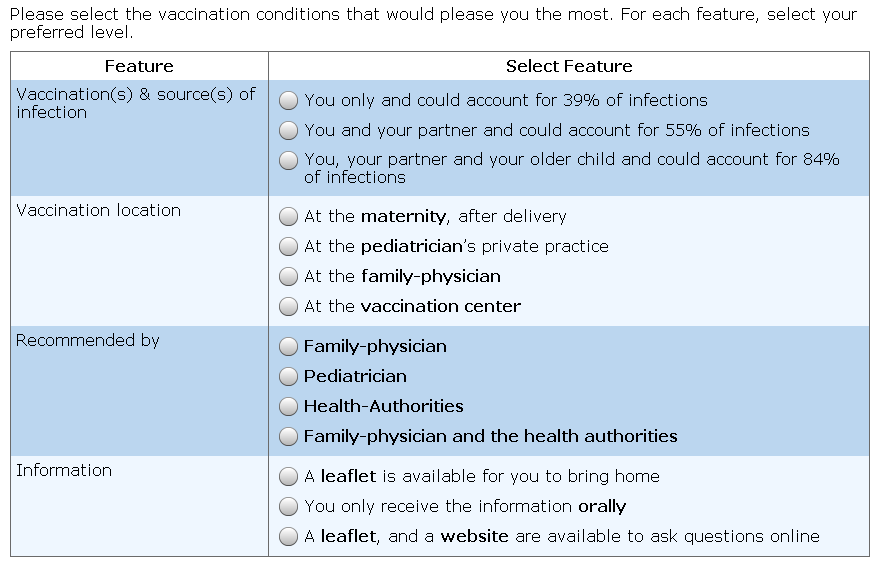


**2) Identify non-compensatory behavior**

**2a) Screening for non-compensatory behavior**


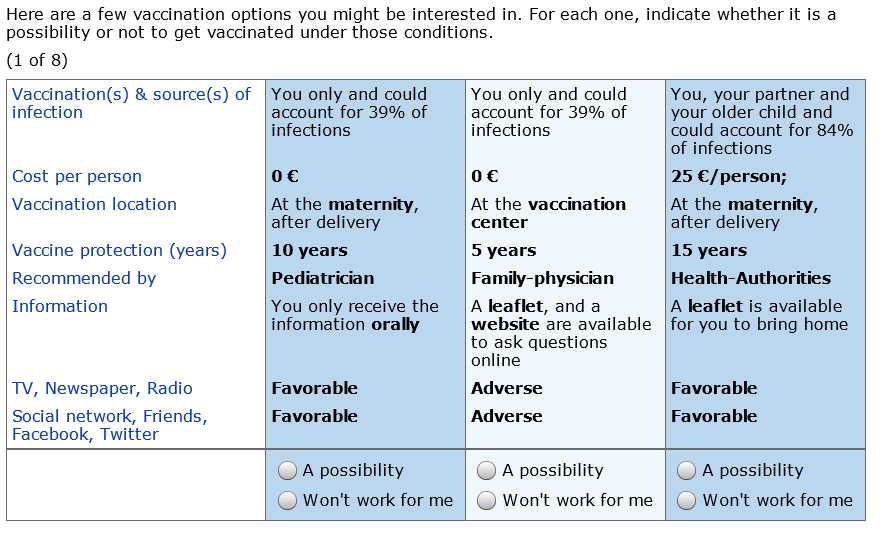


**2b) Confirming the identified behavior as potentially non-acceptable**


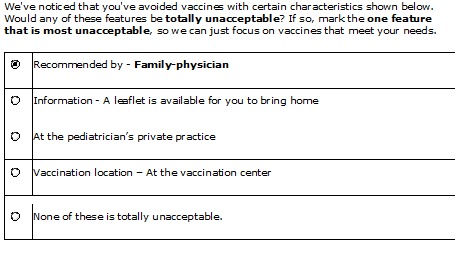


**3) Standard discrete choice experiment part of adaptive (ADCE) questionnaire**


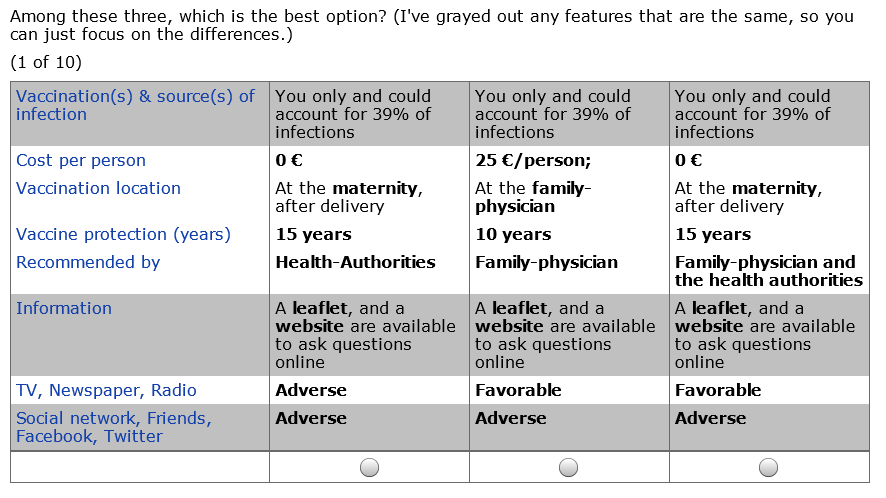


**4) Assess subject’s subjective opinion on the likelihood of adapting vaccination**

**
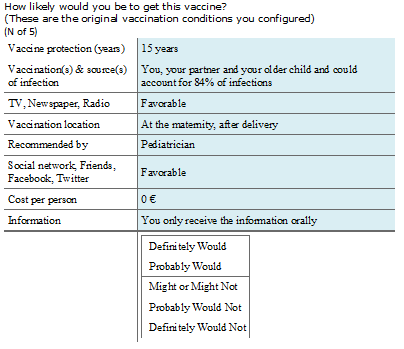
**
