# Supplementary material for: Attributes influencing parental decision-making to receive the Tdap vaccine to reduce the risk of pertussis transmission to their newborn – outcome of a cross-sectional conjoint experiment in Spain and Italy
Source: Hum Vaccin Immunother. 2019 Apr 15;15(5):1080–91. doi: 10.1080/21645515.2019.1571890 (PMC6605846; doi:10.1080/21645515.2019.1571890)
Supplement: Supplemental Material [file khvi-15-05-1571890-s001.zip › Supplementary Figure 3.docx]

# **Supplementary Figure 3. Probability of adopting vaccination at no cost (A) and at 25€/person cost**


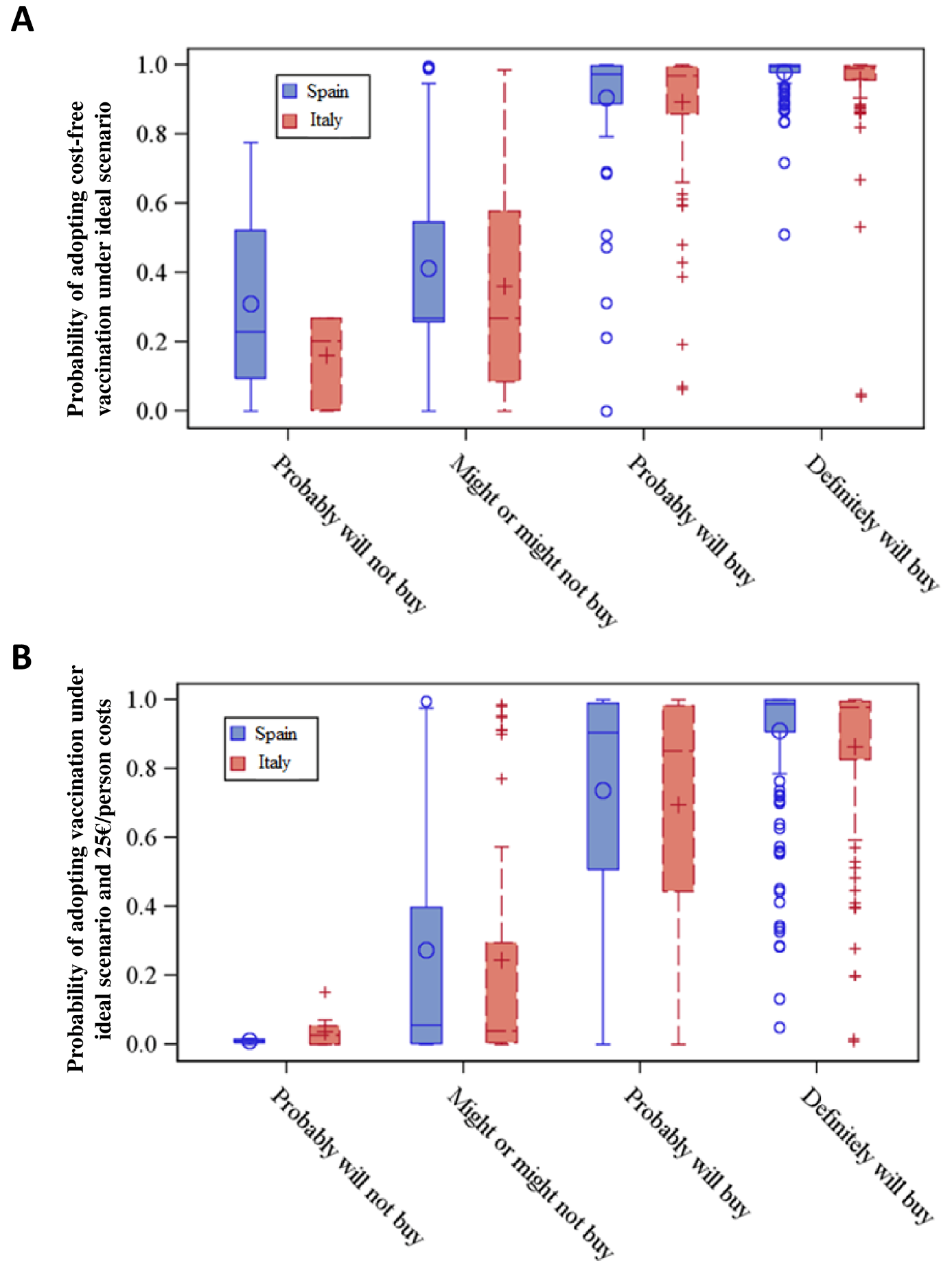


The distribution of the individual probability of adopting vaccination is presented by country for each category of the Likert-scale used in the survey to capture the opinion of the subjects on the likelihood of vaccine adoption. A logistic curve is calibrated to these data to calculate the probability of adoption. The ‘Definitely will not buy’ category included in Supplementary Table 5 is not presented graphically as the number of subjects contributing to that category was not sufficient to provide reliable estimates. Figure part A presents the probability of adopting the subject’s preferred vaccination scenario at no cost versus a no vaccination scenario. Figure part B presents the probability of adopting the subject’s preferred vaccination scenario at a cost of 25€ per person versus a no vaccination scenario.

The limits of the boxes represent the 1^st^ and 3^rd^ quantiles and the bars in the middle represent the medians. The symbols within the box represent the means. The whiskers around the boxes extend up to 1.5 times the interquartile ranges. All extreme observations are shown using symbols beyond the whiskers.
