# Supplementary material for: Attributes influencing parental decision-making to receive the Tdap vaccine to reduce the risk of pertussis transmission to their newborn – outcome of a cross-sectional conjoint experiment in Spain and Italy
Source: Hum Vaccin Immunother. 2019 Apr 15;15(5):1080–91. doi: 10.1080/21645515.2019.1571890 (PMC6605846; doi:10.1080/21645515.2019.1571890)
Supplement: Supplemental Material [file khvi-15-05-1571890-s001.zip › Supplementary Materials.pdf]

## Supplementary Figure 1. Assessment of co-linearity

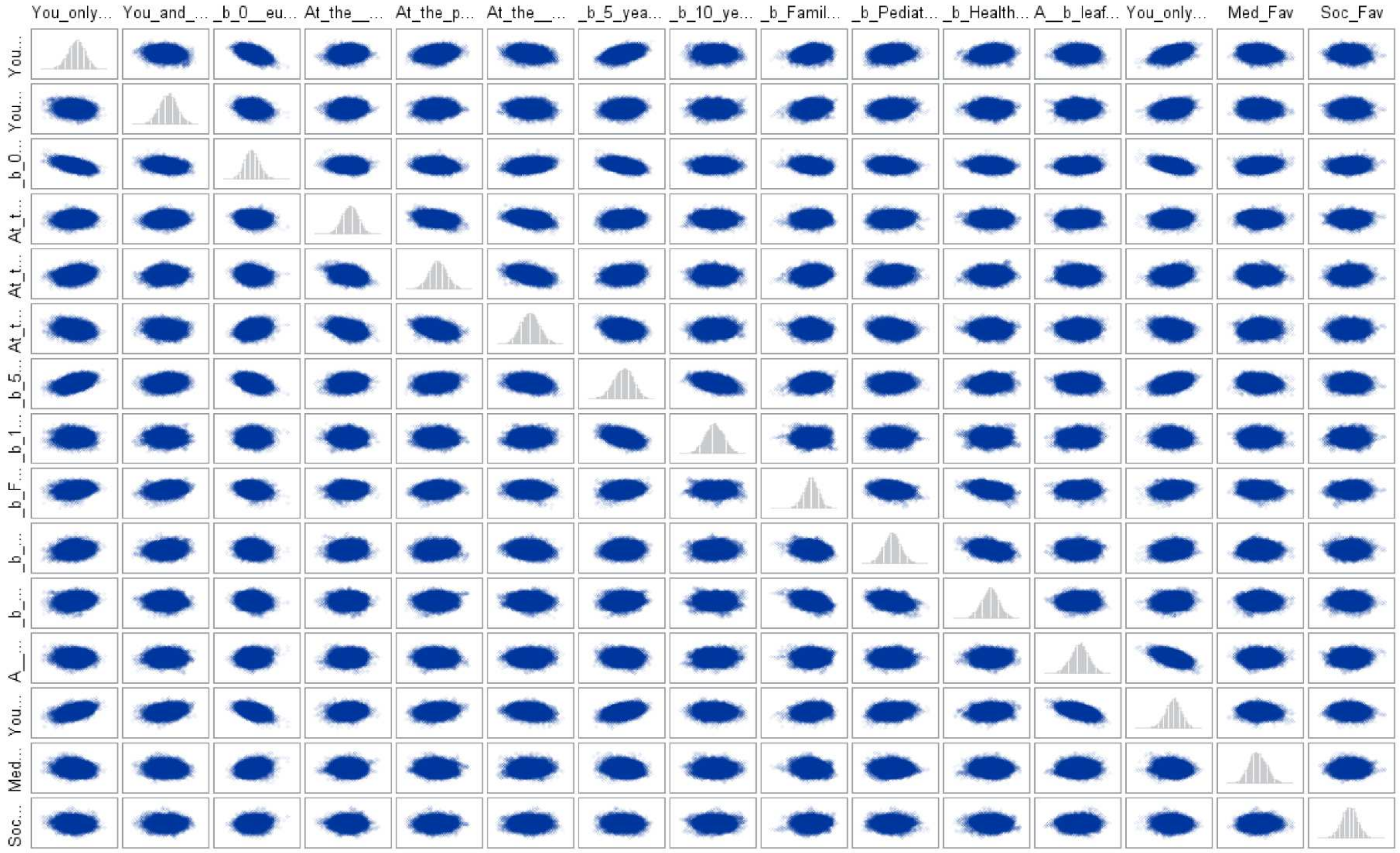


*Correlations between parameters of the hierarchical Bayesian multinomial logit model*

Diagnostic plot illustrating the absence of any relevant dependencies or co-linearity between part-worth utilities. The off-diagonal cells present the bivariate distribution of the x-axis and y-axis parameters included in the model. The diagonal histograms provide insights on the marginal distribution of each parameter. None of those bivariate distributions show extreme dependence as a consequence of co-linearity. Some bivariate plots present some level of correlations between the 2 parameters but exclude any co-linearity.

**Supplementary Figure 2. Examples of a survey using the adaptive discrete choice experiment (ADCE) method**

**1) Identify the local maximum utility scenario**

Please select the vaccination conditions that would please you the most. For each feature, select your preferred level.

| Feature | Select Feature |
|---|---|
| Vaccination(s) & source(s) of infection | ◯ You only and could account for 39% of infections<br>◯ You and your partner and could account for 55% of infections<br>◯ You, your partner and your older child and could account for 84% of infections |
| Vaccination location | ◯ At the **maternity**, after delivery<br>◯ At the **pediatrician**'s private practice<br>◯ At the **family-physician**<br>◯ At the **vaccination center** |
| Recommended by | ◯ **Family-physician**<br>◯ **Pediatrician**<br>◯ **Health-Authorities**<br>◯ **Family-physician and the health authorities** |
| Information | ◯ A **leaflet** is available for you to bring home<br>◯ You only receive the information **orally**<br>◯ A **leaflet**, and a **website** are available to ask questions online |

## 2) Identify non-compensatory behavior

### 2a) Screening for non-compensatory behavior

Here are a few vaccination options you might be interested in. For each one, indicate whether it is a possibility or not to get vaccinated under those conditions.

(1 of 8)

| | | | |
|---|---|---|---|
| Vaccination(s) & source(s) of infection | You only and could account for 39% of infections | You only and could account for 39% of infections | You, your partner and your older child and could account for 84% of infections |
| Cost per person | **0 €** | **0 €** | **25 €/person;** |
| Vaccination location | At the **maternity**, after delivery | At the **vaccination center** | At the **maternity**, after delivery |
| Vaccine protection (years) | **10 years** | **5 years** | **15 years** |
| Recommended by | **Pediatrician** | **Family-physician** | **Health-Authorities** |
| Information | You only receive the information **orally** | A **leaflet**, and a **website** are available to ask questions online | A **leaflet** is available for you to bring home |
| TV, Newspaper, Radio | **Favorable** | **Adverse** | **Favorable** |
| Social network, Friends, Facebook, Twitter | **Favorable** | **Adverse** | **Favorable** |
| | ○ A possibility<br>○ Won't work for me | ○ A possibility<br>○ Won't work for me | ○ A possibility<br>○ Won't work for me |

**2b) Confirming the identified behavior as potentially non-acceptable**

We've noticed that you've avoided vaccines with certain characteristics shown below. Would any of these features be **totally unacceptable**? If so, mark the **one feature that is most unacceptable**, so we can just focus on vaccines that meet your needs.

| | |
|---|---|
| ◉ | Recommended by - **Family-physician** |
| ○ | Information - A leaflet is available for you to bring home |
| ○ | At the pediatrician's private practice |
| ○ | Vaccination location – At the vaccination center |
| ○ | None of these is totally unacceptable. |

**3) Standard discrete choice experiment part of adaptive (ADCE) questionnaire**

Among these three, which is the best option? (I've grayed out any features that are the same, so you can just focus on the differences.)

(1 of 10)

| | | | |
|---|---|---|---|
| Vaccination(s) & source(s) of infection | You only and could account for 39% of infections | You only and could account for 39% of infections | You only and could account for 39% of infections |
| Cost per person | **0 €** | **25 €/person;** | **0 €** |
| Vaccination location | At the **maternity**, after delivery | At the **family-physician** | At the **maternity**, after delivery |
| Vaccine protection (years) | **15 years** | **10 years** | **15 years** |
| Recommended by | **Health-Authorities** | **Family-physician** | **Family-physician and the health authorities** |
| Information | A **leaflet**, and a **website** are available to ask questions online | A **leaflet**, and a **website** are available to ask questions online | A **leaflet**, and a **website** are available to ask questions online |
| TV, Newspaper, Radio | **Adverse** | **Favorable** | **Favorable** |
| Social network, Friends, Facebook, Twitter | **Adverse** | **Adverse** | **Adverse** |
| | ○ | ○ | ○ |

**4) Assess subject's subjective opinion on the likelihood of adapting vaccination**

How likely would you be to get this vaccine?
(These are the original vaccination conditions you configured)
(N of 5)

| | |
|---|---|
| Vaccine protection (years) | 15 years |
| Vaccination(s) & source(s) of infection | You, your partner and your older child and could account for 84% of infections |
| TV, Newspaper, Radio | Favorable |
| Vaccination location | At the maternity, after delivery |
| Recommended by | Pediatrician |
| Social network, Friends, Facebook, Twitter | Favorable |
| Cost per person | 0 € |
| Information | You only receive the information orally |

- Definitely Would
- Probably Would
- Might or Might Not
- Probably Would Not
- Definitely Would Not

## Supplementary Table 1. Relative importance of attributes in Spanish and Italian participants

| Attribute | Relative Importance[a,b] (%) | | | |
|---|---|---|---|---|
| | Spain | | Italy | |
| | Median | Mean | Median | Mean |
| Vaccination(s) & source(s) of infection | 23.06 | 24.04 | 12.86 | 13.91 |
| Cost per person | 11.00 | 13.75 | 14.02 | 15.89 |
| Vaccination location | 13.49 | 14.65 | 18.79 | 20.41 |
| Vaccine protection (years) | 10.49 | 11.07 | 8.39 | 9.56 |
| Recommended by | 13.29 | 13.79 | 15.07 | 16.39 |
| Information | 14.14 | 14.89 | 13.31 | 13.99 |
| TV, Newspaper, Radio | 3.34 | 3.97 | 4.52 | 5.21 |
| Social network, Friends, Facebook, Twitter | 3.07 | 3.85 | 3.51 | 4.63 |

[a] Data represents median and mean relative importance values across respondents for each attribute.
[b] The sum of the mean relative importance values across all attributes equals 100%.

**Supplementary Table 2. Ranking of attribute preferences for the three most important attributes by participants in Spain and Italy**

| Attribute | Order of attribute preference (%) | | | | | |
|---|---|---|---|---|---|---|
| | 1 | | 2 | | 3 | |
| | Spain | Italy | Spain | Italy | Spain | Italy |
| Vaccination(s) & source(s) of infection | 50.2 | 9.9 | 20.1 | 14.6 | 10.9 | 17.9 |
| Cost per person | 19.5 | 26.8 | 13.7 | 11.9 | 8.6 | 11.3 |
| Vaccination location | 10.2 | 28.8 | 17.6 | 31.8 | 19.5 | 15.9 |
| Vaccine protection (years) | 2.2 | 3.6 | 10.2 | 6.0 | 15.3 | 12.6 |
| Recommended by | 7.3 | 14.2 | 18.2 | 21.9 | 20.1 | 22.8 |
| Information | 10.2 | 14.9 | 18.8 | 12.3 | 24.9 | 16.2 |
| TV, Newspaper, Radio | 0.0 | 0.3 | 1.0 | 0.7 | 0.6 | 1.7 |
| Social network, Friends, Facebook, Twitter | 0.3 | 1.3 | 0.3 | 1.0 | 0.0 | 1.7 |

The proportions indicate which attribute was identified as first (1), second (2) or third (3) most important among Spanish or Italian respondents, based on the respondents' individual relative importance (Bayesian estimates) derived from the hierarchical multinomial logit model, using all survey data combined.

**Supplementary Table 3. Ranking of attribute preferences for the three most important attributes by participants in Spain and Italy by gender**

| Attribute | Order of attribute preference (%) | | | | | | Order of attribute preference (%) | | | | | |
|---|---|---|---|---|---|---|---|---|---|---|---|---|
| | Spain | | | | | | Italy | | | | | |
| | 1 | | 2 | | 3 | | 1 | | 2 | | 3 | |
| | Male | Female | Male | Female | Male | Female | Male | Female | Male | Female | Male | Female |
| Vaccination(s) & source(s) of infection | 48.1 | 52.2 | 21.8 | 18.5 | 10.9 | 10.8 | 13.8 | 8.9 | 10.8 | 15.6 | 16.9 | 18.1 |
| Cost per person | 19.2 | 19.7 | 15.4 | 12.1 | 6.4 | 10.8 | 32.3 | 25.3 | 13.8 | 11.4 | 7.7 | 12.2 |
| Vaccination location | 9.0 | 11.5 | 18.6 | 16.6 | 17.9 | 21.0 | 30.8 | 28.3 | 32.3 | 31.6 | 16.9 | 15.6 |
| Vaccine protection (years) | 1.9 | 2.5 | 11.5 | 8.9 | 14.1 | 16.6 | 4.6 | 3.4 | 9.2 | 5.1 | 4.6 | 14.8 |
| Recommended by | 6.4 | 8.3 | 16.0 | 20.4 | 25.6 | 14.6 | 6.2 | 16.5 | 23.1 | 21.5 | 32.3 | 20.3 |
| Information | 15.4 | 5.1 | 15.4 | 22.3 | 23.7 | 26.1 | 10.8 | 16.0 | 9.2 | 13.1 | 18.5 | 15.6 |
| TV, Newspaper, Radio | 0.0 | 0.0 | 0.6 | 1.3 | 1.3 | 0.0 | 0.0 | 0.4 | 1.5 | 0.4 | 1.5 | 1.7 |
| Social network, Friends, Facebook, Twitter | 0.0 | 0.6 | 0.6 | 0.0 | 0.0 | 0.0 | 1.5 | 1.3 | 0.0 | 1.3 | 1.5 | 1.7 |

The proportions indicate which attribute was identified as first (1), second (2) or third (3) most important among Spanish or Italian respondents, based on the respondents' relative importance (Bayesian estimates) derived from the hierarchical multinomial logit model, using all survey data combined. Differences between gender was non-significant (by $Chi^2$ test) in both Spain (p=0.08) and Italy (p=0.21).

## Supplementary Table 4. Relative (part-worth) utilities and approximated p-values of difference with the lowest level for each attribute

| Attribute | Level | Mean part-worth utility | | Utility difference versus the lowest | | 2-sided p-value | | Adjusted p-value[a] | |
|---|---|---|---|---|---|---|---|---|---|
| | | Spain | Italy | Spain | Italy | Spain | Italy | Spain | Italy |
| Vaccination(s) & source(s) of infection | You only and could account for 39% of infections | -71.7 | -28.7 | | | | | | |
| | You and your partner and could account for 55% of infections | -18.1 | 7.8 | 53.6 | 36.5 | <0.0001 | <0.0001 | <0.0001 | <0.0001 |
| | You, your partner and your older child and could account for 84% of infections | 89.8 | 20.9 | 161.5 | 49.6 | <0.0001 | <0.0001 | <0.0001 | <0.0001 |
| Cost per person | 25 euro | -47.4 | -58.1 | | | | | | |
| | 0 euro | 47.4 | 58.1 | 94.8 | 116.2 | <0.0001 | <0.0001 | <0.0001 | <0.0001 |
| Vaccination location[b] | At the pediatrician's private practice | -18.4 | -23.5 | | 19.3 | | <0.0001 | | <0.0001 |
| | At the maternity, after delivery | -5.9 | -42.8 | 12.6 | | 0.0010 | | 0.0029 | |
| | At the vaccination center | 6.8 | 84.4 | 25.2 | 127.2 | <0.0001 | <0.0001 | <0.0001 | <0.0001 |
| | At the family physician | 17.5 | -18.1 | 36.0 | 24.7 | <0.0001 | <0.0001 | <0.0001 | <0.0001 |
| Vaccine protection (years) | 5 | -38.9 | -29.6 | | | | | | |
| | 10 | -0.3 | 3.7 | 38.7 | 33.2 | <0.0001 | <0.0001 | <0.0001 | <0.0001 |
| | 15 | 39.2 | 25.9 | 78.1 | 55.5 | <0.0001 | <0.0001 | <0.0001 | <0.0001 |
| Recommended by | Family physician | -20.5 | -18.5 | | | | | | |
| | Pediatrician | -12.2 | -2.6 | 8.3 | 15.9 | 0.0099 | 0.0002 | 0.0297 | 0.0006 |
| | Health authorities | -7.0 | -9.5 | 13.5 | 9.0 | <0.0001 | 0.0175 | <0.0001 | 0.0526 |
| | Family physician and the health authorities | 39.7 | 30.7 | 60.2 | 49.2 | <0.0001 | <0.0001 | <0.0001 | <0.0001 |
| Information | You only receive the information orally | -48.4 | -41.6 | | | | | | |
| | Printed leaflet is available to bring home | 1.6 | 13.8 | 49.9 | 55.4 | <0.0001 | <0.0001 | <0.0001 | <0.0001 |
| | Printed leaflet and a website to ask questions online | 46.8 | 27.8 | 95.1 | 69.4 | <0.0001 | <0.0001 | <0.0001 | <0.0001 |
| TV, Newspaper, Radio | Adverse | -11.0 | -16.7 | | | | | | |
| | Favorable | 11.0 | 16.7 | 22.0 | 33.5 | <0.0001 | <0.0001 | <0.0001 | <0.0001 |
| Social network, Friends, Facebook, Twitter | Adverse | -7.2 | -5.4 | | | | | | |
| | Favorable | 7.2 | 5.4 | 14.4 | 10.8 | <0.0001 | <0.0001 | <0.0001 | <0.0001 |

[a] 2-sided p-value adjusted for Bonferroni correction for multiple comparisons.
[b] For 'Vaccination location', the reference level representing the lowest preference is different for Spain and Italy.

## Supplementary Table 5. Impact of price on the probability of vaccination adoption

| Scenario | Country | Definitely will not buy | Probably will not buy | Might or might not buy | Probably will buy | Definitely will buy |
|---|---|---|---|---|---|---|
| Probability of vaccination adoption under the participant's most preferred scenario (zero cost) | Spain | 52.2% | 31.0% | 41.2% | 90.5% | 97.7% |
| | Italy | 70.0% | 16.2% | 36.1% | 89.3% | 95.8% |
| Probability of vaccination adoption under the participant's most preferred scenario except price (25€/person) | Spain | 40.8% | 1.0% | 27.4% | 73.7% | 91.0% |
| | Italy | 44.3% | 3.7% | 24.4% | 69.4% | 86.4% |
| Differences between the 2 scenarios | Spain | 11.4% | 30.0% | 13.8% | 16.8% | 6.7% |
| | Italy | 25.7% | 12.5% | 11.7% | 19.9% | 9.4% |

Data represents the mean probability (%) of vaccine adoption based on part-worth utilities and calibration threshold.
A logistic model was calibrated to the data reporting on the opinions of the respondents on vaccine adoption under their most preferred scenario (Table 4) and assuming a 50% probability of vaccination adoption for a level at the Likert-scale at the mid-point between 'Might or might not buy' and 'Probably will buy'. From this model, the values shown reflect the probability of vaccination adoption for each category of this Likert-scale.

**Supplementary Figure 3. Probability of adopting vaccination at no cost (A) and at 25€/person cost**

**A**

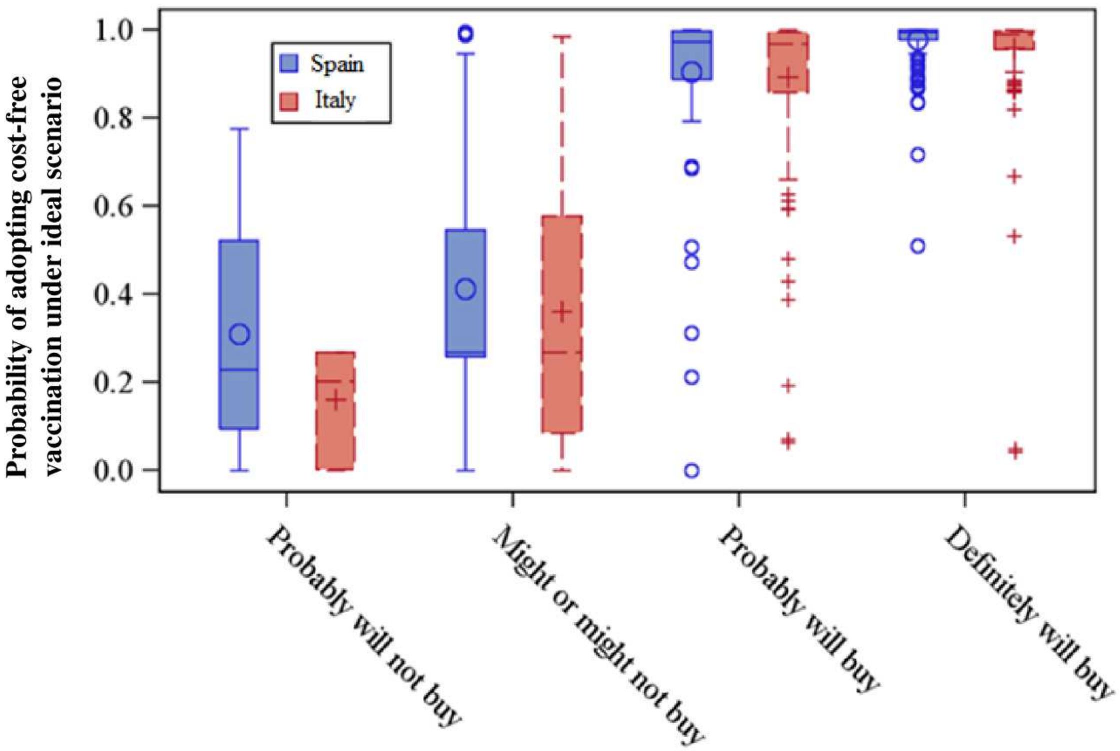


**B**

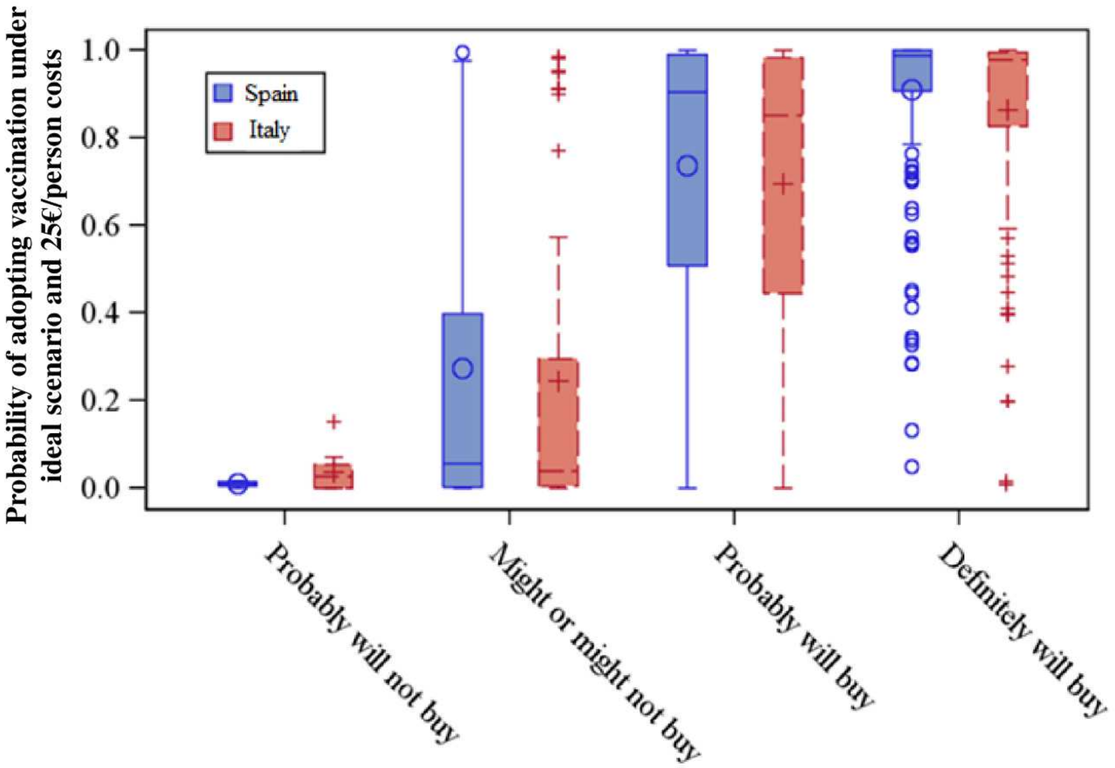


The distribution of the individual probability of adopting vaccination is presented by country for each category of the Likert-scale used in the survey to capture the opinion of the subjects on the likelihood of vaccine adoption. A logistic curve is calibrated to these data to calculate the probability of adoption.

The ‘Definitely will not buy’ category included in Supplementary Table 5 is not presented graphically as the number of subjects contributing to that category was not sufficient to provide reliable estimates. Figure part A presents the probability of adopting the subject’s preferred vaccination scenario at no cost versus a no vaccination scenario. Figure part B presents the probability of adopting the subject’s preferred vaccination scenario at a cost of 25€ per person versus a no vaccination scenario.
The limits of the boxes represent the 1$^{st}$ and 3$^{rd}$ quantiles and the bars in the middle represent the medians. The symbols within the box represent the means. The whiskers around the boxes extend up to 1.5 times the interquartile ranges. All extreme observations are shown using symbols beyond the whiskers.
