# Supplementary material for: Attributes influencing parental decision-making to receive the Tdap vaccine to reduce the risk of pertussis transmission to their newborn – outcome of a cross-sectional conjoint experiment in Spain and Italy
Source: Hum Vaccin Immunother. 2019 Apr 15;15(5):1080–91. doi: 10.1080/21645515.2019.1571890 (PMC6605846; doi:10.1080/21645515.2019.1571890)
Supplement: Supplemental Material [file khvi-15-05-1571890-s001.zip › Supplementary Table 1.docx]

# **Supplementary Table 1. Relative importance of attributes in Spanish and Italian participants**

| **Attribute** | **Relative Importance**^a,b^ (%) | | | |
| --- | --- | --- | --- | --- |
|  | **Spain** | | **Italy** | |
|  | **Median** | **Mean** | **Median** | **Mean** |
| Vaccination(s) & source(s) of infection | 23.06 | 24.04 | 12.86 | 13.91 |
| Cost per person | 11.00 | 13.75 | 14.02 | 15.89 |
| Vaccination location | 13.49 | 14.65 | 18.79 | 20.41 |
| Vaccine protection (years) | 10.49 | 11.07 | 8.39 | 9.56 |
| Recommended by | 13.29 | 13.79 | 15.07 | 16.39 |
| Information | 14.14 | 14.89 | 13.31 | 13.99 |
| TV, Newspaper, Radio | 3.34 | 3.97 | 4.52 | 5.21 |
| Social network, Friends, Facebook, Twitter | 3.07 | 3.85 | 3.51 | 4.63 |

^a^ Data represents median and mean relative importance values across respondents for each attribute.

^b^ The sum of the mean relative importance values across all attributes equals 100%.
