# Supplementary material for: Attributes influencing parental decision-making to receive the Tdap vaccine to reduce the risk of pertussis transmission to their newborn – outcome of a cross-sectional conjoint experiment in Spain and Italy
Source: Hum Vaccin Immunother. 2019 Apr 15;15(5):1080–91. doi: 10.1080/21645515.2019.1571890 (PMC6605846; doi:10.1080/21645515.2019.1571890)
Supplement: Supplemental Material [file khvi-15-05-1571890-s001.zip › Supplementary Table 2.docx]

# **Supplementary Table 2. Ranking of attribute preferences for the three most important attributes by participants in Spain and Italy**

| **Attribute** | **Order of attribute preference (%)** | | | | | |
| --- | --- | --- | --- | --- | --- | --- |
|  | **1** | | **2** | | **3** | |
|  | **Spain** | **Italy** | **Spain** | **Italy** | **Spain** | **Italy** |
| Vaccination(s) & source(s) of infection | 50.2 | 9.9 | 20.1 | 14.6 | 10.9 | 17.9 |
| Cost per person | 19.5 | 26.8 | 13.7 | 11.9 | 8.6 | 11.3 |
| Vaccination location | 10.2 | 28.8 | 17.6 | 31.8 | 19.5 | 15.9 |
| Vaccine protection (years) | 2.2 | 3.6 | 10.2 | 6.0 | 15.3 | 12.6 |
| Recommended by | 7.3 | 14.2 | 18.2 | 21.9 | 20.1 | 22.8 |
| Information | 10.2 | 14.9 | 18.8 | 12.3 | 24.9 | 16.2 |
| TV, Newspaper, Radio | 0.0 | 0.3 | 1.0 | 0.7 | 0.6 | 1.7 |
| Social network, Friends, Facebook, Twitter | 0.3 | 1.3 | 0.3 | 1.0 | 0.0 | 1.7 |

The proportions indicate which attribute was identified as first (1), second (2) or third (3) most important among Spanish or Italian respondents, based on the respondents’ individual relative importance (Bayesian estimates) derived from the hierarchical multinomial logit model, using all survey data combined.
