# Supplementary material for: Attributes influencing parental decision-making to receive the Tdap vaccine to reduce the risk of pertussis transmission to their newborn – outcome of a cross-sectional conjoint experiment in Spain and Italy
Source: Hum Vaccin Immunother. 2019 Apr 15;15(5):1080–91. doi: 10.1080/21645515.2019.1571890 (PMC6605846; doi:10.1080/21645515.2019.1571890)
Supplement: Supplemental Material [file khvi-15-05-1571890-s001.zip › Supplementary Table 4.docx]

# **Supplementary Table 4. Relative (part-worth) utilities and approximated p-values of difference with the lowest level for each attribute**

| **Attribute** | **Level** | **Mean part-worth utility** | | **Utility difference versus the lowest** | | **2-sided p-value** | | **Adjusted p-value**^a^ | |
| --- | --- | --- | --- | --- | --- | --- | --- | --- | --- |
|  |  | **Spain** | **Italy** | **Spain** | **Italy** | **Spain** | **Italy** | **Spain** | **Italy** |
| Vaccination(s) & source(s) of infection | You only and could account for 39% of infections | -71.7 | -28.7 |  |  |  |  |  |  |
|  | You and your partner and could account for 55% of infections | -18.1 | 7.8 | 53.6 | 36.5 | <0.0001 | <0.0001 | <0.0001 | <0.0001 |
|  | You, your partner and your older child and could account for 84% of infections | 89.8 | 20.9 | 161.5 | 49.6 | <0.0001 | <0.0001 | <0.0001 | <0.0001 |
| Cost per person | 25 euro | -47.4 | -58.1 |  |  |  |  |  |  |
|  | 0 euro | 47.4 | 58.1 | 94.8 | 116.2 | <0.0001 | <0.0001 | <0.0001 | <0.0001 |
| Vaccination location^b^ | At the pediatrician’s private practice | -18.4 | -23.5 |  | 19.3 |  | <0.0001 |  | <0.0001 |
|  | At the maternity, after delivery | -5.9 | -42.8 | 12.6 |  | 0.0010 |  | 0.0029 |  |
|  | At the vaccination center | 6.8 | 84.4 | 25.2 | 127.2 | <0.0001 | <0.0001 | <0.0001 | <0.0001 |
|  | At the family physician | 17.5 | -18.1 | 36.0 | 24.7 | <0.0001 | <0.0001 | <0.0001 | <0.0001 |
| Vaccine protection (years) | 5 | -38.9 | -29.6 |  |  |  |  |  |  |
|  | 10 | -0.3 | 3.7 | 38.7 | 33.2 | <0.0001 | <0.0001 | <0.0001 | <0.0001 |
|  | 15 | 39.2 | 25.9 | 78.1 | 55.5 | <0.0001 | <0.0001 | <0.0001 | <0.0001 |
| Recommended by | Family physician | -20.5 | -18.5 |  |  |  |  |  |  |
|  | Pediatrician | -12.2 | -2.6 | 8.3 | 15.9 | 0.0099 | 0.0002 | 0.0297 | 0.0006 |
|  | Health authorities | -7.0 | -9.5 | 13.5 | 9.0 | <0.0001 | 0.0175 | <0.0001 | 0.0526 |
|  | Family physician and the health authorities | 39.7 | 30.7 | 60.2 | 49.2 | <0.0001 | <0.0001 | <0.0001 | <0.0001 |
| Information | You only receive the information orally | -48.4 | -41.6 |  |  |  |  |  |  |
|  | Printed leaflet is available to bring home | 1.6 | 13.8 | 49.9 | 55.4 | <0.0001 | <0.0001 | <0.0001 | <0.0001 |
|  | Printed leaflet and a website to ask questions online | 46.8 | 27.8 | 95.1 | 69.4 | <0.0001 | <0.0001 | <0.0001 | <0.0001 |
| TV, Newspaper, Radio | Adverse | -11.0 | -16.7 |  |  |  |  |  |  |
|  | Favorable | 11.0 | 16.7 | 22.0 | 33.5 | <0.0001 | <0.0001 | <0.0001 | <0.0001 |
| Social network, Friends, Facebook, Twitter | Adverse | -7.2 | -5.4 |  |  |  |  |  |  |
|  | Favorable | 7.2 | 5.4 | 14.4 | 10.8 | <0.0001 | <0.0001 | <0.0001 | <0.0001 |

^a^ 2-sided p-value adjusted for Bonferroni correction for multiple comparisons.

^b^ For ‘Vaccination location’, the reference level representing the lowest preference is different for Spain and Italy.
