# Supplementary material for: Attributes influencing parental decision-making to receive the Tdap vaccine to reduce the risk of pertussis transmission to their newborn – outcome of a cross-sectional conjoint experiment in Spain and Italy
Source: Hum Vaccin Immunother. 2019 Apr 15;15(5):1080–91. doi: 10.1080/21645515.2019.1571890 (PMC6605846; doi:10.1080/21645515.2019.1571890)
Supplement: Supplemental Material [file khvi-15-05-1571890-s001.zip › Supplementary Table 5.docx]

# **Supplementary Table 5. Impact of price on the probability of vaccination adoption**

| **Scenario** | **Country** | **Definitely will not buy** | **Probably will not buy** | **Might or might not buy** | **Probably will buy** | **Definitely will buy** |
| --- | --- | --- | --- | --- | --- | --- |
| Probability of vaccination adoption under the participant’s most preferred scenario (zero cost) | Spain | 52.2% | 31.0% | 41.2% | 90.5% | 97.7% |
|  | Italy | 70.0% | 16.2% | 36.1% | 89.3% | 95.8% |
| Probability of vaccination adoption under the participant’s most preferred scenario except price (25€/person) | Spain | 40.8% | 1.0% | 27.4% | 73.7% | 91.0% |
|  | Italy | 44.3% | 3.7% | 24.4% | 69.4% | 86.4% |
| Differences between the 2 scenarios | Spain | 11.4% | 30.0% | 13.8% | 16.8% | 6.7% |
|  | Italy | 25.7% | 12.5% | 11.7% | 19.9% | 9.4% |

Data represents the mean probability (%) of vaccine adoption based on part-worth utilities and calibration threshold.

A logistic model was calibrated to the data reporting on the opinions of the respondents on vaccine adoption under their most preferred scenario (Table 4) and assuming a 50% probability of vaccination adoption for a level at the Likert-scale at the mid-point between ‘Might or might not buy’ and ‘Probably will buy’. From this model, the values shown reflect the probability of vaccination adoption for each category of this Likert-scale.
